# A Case of Spondylodysplastic Ehlers-Danlos Syndrome With Comorbid Hypophosphatasia

**DOI:** 10.1016/j.aace.2022.08.005

**Published:** 2022-09-02

**Authors:** Antara Dattagupta, Shelley Williamson, Lamees I. El Nihum, Steven Petak

**Affiliations:** 1 Division of Endocrinology, Diabetes, and Metabolism, Department of Medicine, Houston Methodist Hospital, Houston, Texas; 2 Texas A&M College of Medicine, Texas A&M University, Bryan, Texas

**Keywords:** Ehlers-Danlos syndrome, hypophosphatasia, ALPL, B4GALT7, asfotase alfa, ALP, alkaline phosphatase, EDS, Ehlers-Danlos syndrome, HPP, hypophosphatasia, spEDS, spondylodysplastic Ehlers-Danlos syndrome

## Abstract

**Background/Objective:**

Spondylodysplastic Ehlers-Danlos syndrome (spEDS) is a rare subtype of the heritable connective tissue disorder characterized in the 2017 Ehlers-Danlos syndrome (EDS) nosology. Three biallelic mutations, *B4GALT7*, *B3GALT6*, and *SLC39A13*, confirm the diagnosis of spEDS. Hypophosphatasia (HPP) is a heritable disorder caused by a genetic sequence variation in the *ALPL* gene affecting bone mineralization. Common symptoms in the adult form of HPP are joint pain, muscle hypotonia, and metatarsal fractures. Here we present a case of spEDS and HPP in a patient.

**Case Report:**

A 38-year-old woman was evaluated for chronic diffuse joint pain and a low alkaline phosphatase level of 27 U/L (reference, 31-125 U/L). In addition, she presented with a history of hypermobility, limb bowing, and hyperextensible skin, prompting genetic testing for EDS and HPP. The results returned significant for a synonymous sequence variant at c.441G>A in the *B4GALT7* gene indicative of spEDS. HPP was clinically diagnosed by a repeat low alkaline phosphatase level of 23 U/L and high vitamin B6 level of 24.4 ng/mL (reference, 2.1-21.7 ng/mL), despite the absence of the *ALPL* gene sequence variation on genetic testing.

**Discussion:**

Remarkable personal and family history of this patient suggest that co-occurrence of EDS and HPP is not merely coincidental. Given the overlapping features of muscle hypotonia and joint pain between the 2 heritable disorders, a possible relationship between the 2 may have been previously overlooked.

**Conclusion:**

Further investigation in the relationship and management of the 2 heritable diseases is warranted as enzyme replacement therapy, asfotase alfa, approved for infantile and juvenile onset of HPP may improve the symptoms shared with EDS.

**Highlights**

• Spondylodysplastic Ehlers-Danlos syndrome with comorbid hypophosphatasia
• Unexplored relationship between the B4GALT7 protein and *ALPL* gene
• Suggested expansion of indications for asfotase alfa therapy

**Clinical Relevance**

We describe a novel phenotype of spondylodysplastic Ehlers-Danlos syndrome (spEDS) in a 38-year-old woman with comorbid hypophosphatasia (HPP). spEDS was confirmed through genetic testing. HPP was clinically diagnosed, despite the absence of the *ALPL* gene sequence variation. This co-occurrence warrants further investigation in the relationship between the 2 rare heritable disorders and indications for asfotase alfa therapy.

## Introduction

Ehlers-Danlos syndrome (EDS) refers to a diverse group of heritable connective tissue disorders caused by various genetic sequence variations. Spondylodysplastic EDS (spEDS) is a rare subtype from the 2017 EDS nosology that is caused by 3 biallelic sequence variations, *B4GALT7*, *B3GALT6*, and *SLC39A13*.^1^^,^^2^ Within the nosology, major and minor criteria are shared by the 3 biallelic sequence variations to clinically diagnose spEDS. The gene-specific minor criteria for each biallelic sequence variation aid in differentiation and diagnosis. The gene-specific minor criteria for *B4GALT7* are radioulnar synostosis, bilateral elbow contractures, generalized joint hypermobility, single transverse palmar crease, characteristic craniofacial features, characteristic radiographic findings, severe hypermetropia, and clouded cornea. Minimal criteria to suggest spEDS diagnosis require 2 major criteria and at least 3 minor criteria.^1^ Ultimately, molecular testing confirms the diagnosis.

Hypophosphatasia (HPP) is a rare heritable disorder caused by loss-of-function sequence variation in *ALPL*, which leads to deficient tissue-nonspecific alkaline phosphatase (ALP) that is essential for bone mineralization.^3^ HPP has a wide spectrum of clinical manifestations with severity ranging from perinatal, which can be lethal at birth, to mild symptoms in the adult form.^4^ The symptoms of the adult form of HPP can vary and are often diagnosed after middle age. Generally, these patients experience pain by recurrent metatarsal stress fractures, chronic muscle pain and/or muscular hypotonia, bone and joint pain, seizures, rickets, and dental problems.

There have not been previous cases reported of patients with comorbid spEDS and HPP. With several overlapping features of the 2 rare heritable disorders affecting the muscles, bones, and joints, it is important to note any relationship between the 2 to help determine therapy options for patients. Here, we present a case of spEDS and HPP in a patient who presented with chronic diffuse joint pain and hypermobility.

## Case Report

A 38-year-old woman was referred for evaluation of a low ALP level of 27 U/L (reference, 31-125 U/L) and a 5-year history of diffuse joint pain. Before her referral to endocrinology, she had a normal parathyroid hormone level of 41.30 pg/mL (reference, 14-64 pg/mL) with a calcium level of 9.9 mg/dL (reference, 8.6-10.2 mg/dL) (Table 1). She reported a history of hypermobility in childhood, and previous medical records confirmed a medical history of Gilbert syndrome, vitamin D deficiency, thyroid nodules, essential tremor, and visual impairment. Of note, she underwent transsphenoidal resection of a pituitary cyst in 2004. She weighed 173 pounds, and her height was 5 feet and 6 inches.

**Table 1:** Clinical Outpatient Laboratory Values

Lab reference range	Reference range	Laboratory results 4 y before referral	Laboratory results on referral
Alkaline phosphatase	31-125 U/L	27 (low)	23 (low)
Calcium	8.6-10.2 mg/dL	9.9	10
Phosphorus	2.5-4.5 mg/dL	…	4.3
Alkaline phosphatase, bone specific	5.3-19.5 mcg/L	…	4.8 (low)
Vitamin B6	2.1-21.7 ng/mL	…	24.4 (high)
Parathyroid hormone	14-64 pg/mL	41.30	…

Consistent laboratory results indicated hypophosphatasia because of low alkaline phosphatase and elevated vitamin B6 levels.

The family medical history was remarkable for multiple family members with signs and symptoms of both HPP and EDS. Specifically, both of the patient’s daughters had flat feet, pes planus, and joint hypermobility. Her youngest daughter also exhibited joint pain and bowing of the legs. Similar to our patient, both of her daughters exhibited age-appropriate growth. Neither the patient nor her daughters experienced any motor or cognitive delay in development. Her daughters have not undergone genetic testing at this time but plan to test when they are older. Her mother shares similar characteristics of shoulder and knee hypermobility. Her sister and nephew are currently undergoing evaluation for a different subtype of EDS after having resulted in a confirmed ZNF423 sequence variation suggestive of this heritable disease. Additionally, her father and sister both have a history of low ALP levels with her sister’s most recent ALP level of 28 U/L.

On referral to endocrinology, she noted a history of joint hypermobility, diffuse joint pain, hyperextensible skin, pes planus, limb bowing, piezogenic papules on the medial aspects of her bilateral heels and muscle hypotonia of the lower extremities (Table 2). She also reported a history of multiple finger and toe fractures and kidney stones. Based on her history and physical examination findings, repeat laboratory work, and a dual-energy x-ray absorptiometry scan was ordered with a follow-up appointment in 1 month.

**Table 2:** Patient Evaluation Under Spondylodysplastic Ehlers-Danlos Syndrome Criteria

	Present patient (*spEDS-B4GALT7)*
**Major criteria**	
Short stature	**−**
Muscle hypotonia	+
Bowing of limbs	**+**
**Minor criteria**	
Hyperextensible, soft, doughy, and thin skin	+
Pes planus, equinovarus, or valgus	+
Delayed motor development	−
Low bone mass (osteopenia)	−
Delayed cognitive development	−
***B4GALT7-*****specific minor criteria**	
Radioulnar synostosis	−
Bilateral elbow contractures	−
Generalized joint hypermobility	+
Single transverse palmar crease	−
Characteristic craniofacial features	−
Characteristic radiographic findings	−
Severe hypermetropia	−
Clouded cornea	−

During her follow-up appointment, her dual-energy x-ray absorptiometry showed a bone density within expected range for her age with an anteroposterior spine (L1-L4) z-score of +1.5, left femoral neck z-score of +0.5, right femoral neck z-score of +0.2, total left hip z-score of +1.0, right total hip z-score of +1.4, and trabecular bone score of 1.503. Her 10-year major osteoporotic fracture risk was 1.7% (Table 3). Repeat laboratory results indicated a low ALP level of 23 U/L and high vitamin B6 level of 24.4 ng/mL (reference, 2.1-21.7 ng/mL). Given her history of hypermobility, chronic diffuse joint pain, and laboratory results of low ALP and high vitamin B6 levels, the patient underwent genetic testing for both EDS and HPP, which was performed via saliva swab (PreventionGenetics). The results revealed a heterozygous sequence variation of the *B4GALT7* gene and absence of the *ALPL* gene sequence variation.

**Table 3:** Dual-Energy X-Ray Absorptiometry

Region	Bone mineral density, g/cm^2^	z-score
AP spine (L1-L4)	1.189	1.5
Femoral neck (left)	0.876	0.5
Total hip (left)	1.045	1.0
Femoral neck (right)	0.844	0.2
Total hip (right)	1.100	1.4
Total hip mean	1.073	…
10-y fracture risk	Major osteoporotic fracture: 1.7%	…
	Hip fracture: <0.1%	…

Abbreviation: AP = anteroposterior.

The scan results did not indicate low bone mass (osteopenia).

## Discussion

spEDS-*B4GALT7* is rare with limited literature regarding the heritable disorder. Our patient satisfied 2 of the major criteria, 2 of the minor criteria, and 1 of the gene-specific minor criteria for spEDS- *B4GALT7*. With regard to the major criteria, she reported a history of muscle hypotonia, most notably in her lower extremity. When aligning her feet together, she exhibited bowing of her limbs. With regard to the minor criteria, she described her skin as “stretchy” and her feet as “flat.” She did not experience any motor or cognitive delay in development. In addition to the major and minor criteria associated with spEDS, our patient exhibited a unique presentation of piezogenic papules in the medial aspects of her feet bilaterally. Piezogenic papules occur because of herniation of subcutaneous fat into the dermis, often caused by compressive stress. Although rare, existing literature describes an association between the presence of piezogenic papules and EDS.^5^ Our patient consented to provide photographic verification of the major and minor criteria she exhibited (Fig. *A* through *F*).

**Fig:**
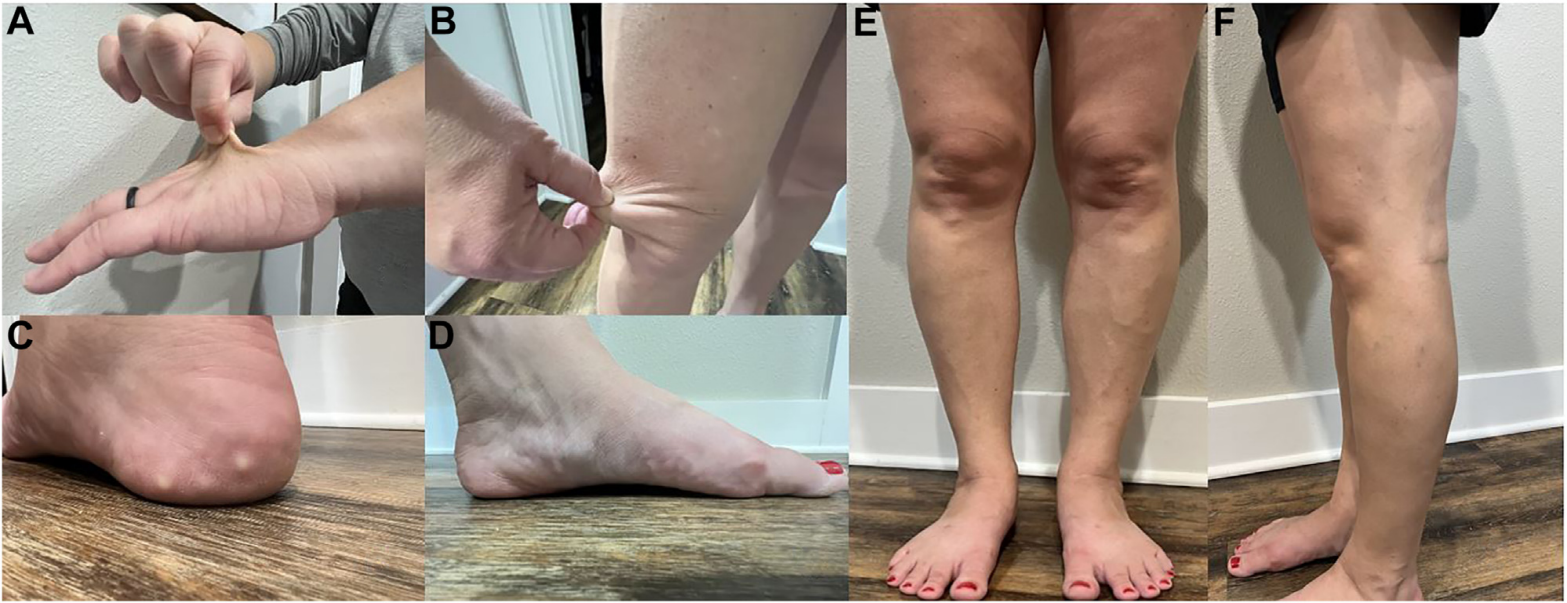
Clinical findings of spondylodysplastic Ehlers-Danlos syndrome. The patient exhibited features of skin hyperextensibility (*A-B*), piezogenic papules (*C*), pes planus (*D*), bowing of the legs (*E*), and muscle hypotonia (*F*).

The results from genetic testing revealed a heterozygous mutation of the *B4GALT7* gene that is inherited in an autosomal recessive pattern. This contains a sequence variant at c.441G>A (Gly441Ala), which is suggested to activate a cryptic donor splice site. Remarkably, this sequence variant has not been reported in current public databases or literature to date. Therefore, this sequence variant is of unknown significance.

Although genetic testing resulted in a negative *ALPL* sequence variation that would have been indicative of HPP diagnosis, our patient was clinically diagnosed with HPP given her symptoms suggestive of the adult form of HPP and laboratory values of chronically low ALP and elevated vitamin B6 levels. No other known causes were shown to be a source of her low ALP levels because her parathyroid hormone, calcium, and phosphorus levels were within the normal range, ruling out other secondary causes, such as hypervitaminosis D.

We hypothesize that the result of the negative *ALPL* sequence variation are because of our patient possibly either having a mutated sequence variant in the *ALPL* gene that was not among the ones selected in this particular genetic test or having another sequence variation potentially related to her spEDS sequence variant causing an HPP-like presentation. Remarkable personal and family histories of this patient support the hypothesis that the co-occurrence of EDS and HPP is not merely coincidental. Our patient is currently undergoing whole genome sequencing to further analyze the cause of her clinical diagnosis of HPP. This investigation will search for cryptic alterations in the *ALPL* gene not identified in commercial testing.

In literature, HPP is frequently misdiagnosed in patients and treated incorrectly with bisphosphonates or high doses of vitamin D and calcium supplementation that potentially complicate and worsen symptoms.^4^ Patients with HPP conveyed a diminished quality of life because of their symptoms related to HPP. Similar to our patient, those with chronic pain in the bones, joints, and muscles have reported the daily use of analgesic medications. Enzyme replacement therapy, asfotase alfa, also known as Strensiq, is a promising therapy for patients with HPP. It has been shown to significantly improve bone mineralization, alleviate bone pain, and increase strength, agility, and motor function.^6^ Although currently only Food and Drug Administration approved for treatment of infantile and juvenile onset of HPP, this therapy could potentially benefit adult patients with a clinical diagnosis of HPP.

With overlapping clinical features of muscle hypotonia and chronic diffuse joint between the 2 rare heritable diseases, further investigation is warranted to determine a relationship between the 2 that may have been overlooked previously. This is important to investigate because the treatment of HPP with asfotase alfa may also improve patients’ comorbid spEDS muscular, bone, and joint symptoms.

## Conclusion

Our patient’s unique presentation of spEDS with comorbid HPP has not been previously noted in literature. We also report a sequence variant for spEDS at c.441G>A (Gly441Ala) in the *B4GALT7* gene that has not been reported in current public databases. Despite having chronically low ALP and high vitamin B6 levels and clinical symptoms consistent with the disease, our patient was clinically diagnosed with HPP given the negative *ALPL* sequence variation on genetic studies and is currently undergoing whole genome sequencing for further analysis. The patient’s extensive family history consistent with both HPP and spEDS features prompts additional investigation to determine a relationship to appropriately manage the shared symptoms between the 2 rare heritable disorders.

## Disclosure

The authors have no multiplicity of interest to disclose. S.P. is a speaker for Alexion.
